# Secreted and Tissue miRNAs as Diagnosis Biomarkers of Malignant Pleural Mesothelioma

**DOI:** 10.3390/ijms19020595

**Published:** 2018-02-17

**Authors:** Vanessa Martínez-Rivera, María Cristina Negrete-García, Federico Ávila-Moreno, Blanca Ortiz-Quintero

**Affiliations:** 1 Research Unit, Instituto Nacional de Enfermedades Respiratorias “Ismael Cosio Villegas”, Calzada de Tlalpan 4502, Colonia Sección XVI, 14080 Mexico City, Mexico; vanessa.m.r.0801@gmail.com (V.M.-R.); cristi.negrete@gmail.com (M.C.N.-G.); 2 Unidad de Investigación en Biomedicina (UBIMED), Cancer Epigenomics and Lung Disease Laboratory 12, Facultad de Estudios Superiores (FES)-Iztacala, Universidad Nacional Autónoma de México, Avenida de los Barrios #1 Colonia los Reyes Iztacala, 54090 Mexico City, Mexico; f.avila@unam.mx

**Keywords:** malignant pleural mesothelioma, microRNAs, diagnosis biomarkers

## Abstract

Malignant pleural mesothelioma (MPM) is a rare but aggressive tumor that originates in the pleura, is diagnosed in advanced stages and has a poor prognosis. Accurate diagnosis of MPM is often difficult and complex, and the gold standard diagnosis test is based on qualitative analysis of markers in pleural tissue by immunohistochemical staining. Therefore, it is necessary to develop quantitative and non-subjective alternative diagnostic tools. MicroRNAs are non-coding RNAs that regulate essential cellular mechanisms at the post-transcriptional level. Recent evidence indicates that miRNA expression in tissue and body fluids is aberrant in various tumors, revealing miRNAs as promising diagnostic biomarkers. This review summarizes evidence regarding secreted and tissue miRNAs as biomarkers of MPM and the biological characteristics associated with their potential diagnostic value. In addition to studies regarding miRNAs with potential diagnostic value for MPM, studies that aimed to identify the miRNAs involved in molecular mechanisms associated with MPM development are described with an emphasis on relevant aspects of the experimental designs that may influence the accuracy, consistency and real diagnostic value of currently reported data.

## 1. Introduction

Malignant pleural mesothelioma (MPM) is an uncommon but aggressive tumor that originates in mesothelial cells of the pleural membrane [1,2]. The disease is associated with asbestos exposure in 80% of cases, and symptoms manifest after a prolonged period of latency after exposure (20–40 years) [2,3,4] with a survival of 9–12 months [1,2,3,5].

The World Health Organization (WHO) estimated 92,252 worldwide deaths in the period of 1994–2008 due to this disease [6]; however, this figure may be underestimated due to the lack of reliable records on MPM diagnosis [7]. Moreover, an increase in the global incidence of mesothelioma is predicted based on high exposure to asbestos in past years [2].

MPM is classified histologically into three types: epithelioid, sarcomatoid and biphasic [5,8]. The epithelioid type is the most frequent with approximately 50–60% of cases [9]. Overall, accurate diagnosis of MPM is considered difficult and complex. First, early clinical MPM symptomatology is not disease-specific; however, advanced stages are characterized by pleural effusion, chest pain and dyspnea [10], which usually lead to chest X-ray analysis. Broad chest X-ray analysis can detect the presence of diffuse pleural thickening and nodular prominences, which suggest mesothelioma [2,11,12]. Cytological examination of the pleural fluid can be performed; however, only 60% of true positive cases can be identified using this technique [2,13,14]. The confirmatory diagnosis test or gold standard is based on the detection of pleural tissue markers by immunohistochemistry [15], but it requires pleural tissue samples obtained by invasive techniques [16]. In addition, there is no known marker that is 100% diagnostic; therefore, a combination of antibodies that recognize several positive and negative markers in MPM is used [8]. A pathologist observes the tissue under a microscope and decides whether the test is positive or negative based on his/her criteria and experience; therefore, diagnosis is subjective and qualitative. For MPM diagnosis, at least two positive and two negative markers are recommended [17]. Typically, MPM is positive for calretinin and cytokeratin 5/6 but negative for thyroid transcription factor 1 (TTF-1) and carcinoembryonic antigen (CEA) [8,17]. In addition, epithelial MPM (tubulopapillary and acinar subtype) may be difficult to distinguish from metastatic lung adenocarcinoma (AD), due to the mesenchymal/epithelial pattern that is present in both [8,17]. Their differential diagnosis requires an additional panel of antibodies in immunohistochemical staining that is positive for epithelial MPM [17]. This differential diagnosis is relevant since the treatment regimen is different for each disease. Because of this complexity, the MPM diagnostic guide issued by the “International Mesothelioma Interest Group (IMIG)” recommends that diagnosis is based on the interpretation of clinical, radiological and pathological findings altogether [17] in order to increase the likelihood of an accurate MPM diagnosis.

Therefore, the development of alternative, quantitative diagnostic biomarkers would have significant clinical potential.

In recent years, microRNAs (miRNAs) have been the subject of intense studies as key regulators of gene expression at the post-transcriptional level. Furthermore, it was found that miRNA expression was altered in tumor tissue and body fluids from several neoplastic pathologies, pointing to miRNAs as potential diagnostic biomarkers. Moreover, the miRNAs found in biological samples have all been stable and quantifiable.

Alternative miRNA-based biomarker tests could add relevant adjunct information and increase the probability of reaching the right diagnosis.

This review aims to summarize evidence regarding the potential diagnostic value of secreted and tissue miRNAs as MPM biomarkers with an emphasis on the relevant aspects of experimental designs that may influence the accuracy, consistency and real diagnostic value of currently reported data.

## 2. MicroRNAs

miRNAs are small double-stranded RNAs of ~22 nt that regulate gene expression at the post-transcriptional level by blocking the translation of target messenger RNA [18]. miRNAs have been found in every organism analyzed to date and regulate essential cellular processes, such as differentiation, proliferation and apoptosis [18,19,20]. It is important to note that these cellular processes are deregulated in several neoplastic processes [21,22]. miRNAs are expressed in normal physiological conditions in a cell- and tissue-specific-manner, but their expression pattern was found to be aberrant in tumor tissue and could be distinguished from the normal expression pattern of healthy tissue. Cumulative evidence showed that altered miRNA expression profiles in tumor tissues could be associated with the diagnosis, prognosis and even histological classification of lung, breast, colorectal, and pancreatic cancer, hepatocellular carcinoma and malignant mesothelioma, among others [23,24,25,26,27,28,29]. Moreover, several of these tissue miRNAs have been associated with carcinogenesis per se: experimental manipulation of certain altered miRNAs in several cancers has been shown to regulate the tumorigenic properties of tumor cell lines and tumor growth in mouse models [30,31,32,33]. In addition, the expression of tissue miRNAs does not seem to depend on the age or race of the individual [34,35].

Nevertheless, tumor tissue sampling requires the use of invasive retrieval techniques. Favorably, cell-free miRNAs are also detected in peripheral blood (circulating miRNAs) and in other body fluids, such as tears, urine, and saliva. In healthy individuals, miRNAs are secreted by cells into body fluids with stable and constant concentrations [36]; however, similar to tissue miRNAs, alterations in their expression levels have been associated with several cancers [36]. These secreted cell-free miRNAs are resistant to endogenous RNases and external conditions, such as freeze-thaw and extreme pH [36,37,38]. These characteristics are desirable in potential diagnostic biomarkers due to their transport, storage and manipulation in the laboratory. Because obtaining peripheral blood and body fluids is less invasive than obtaining tissue samples, secreted miRNAs are considered to be potential non-invasive biomarkers of cancer.

**Figure 1:**
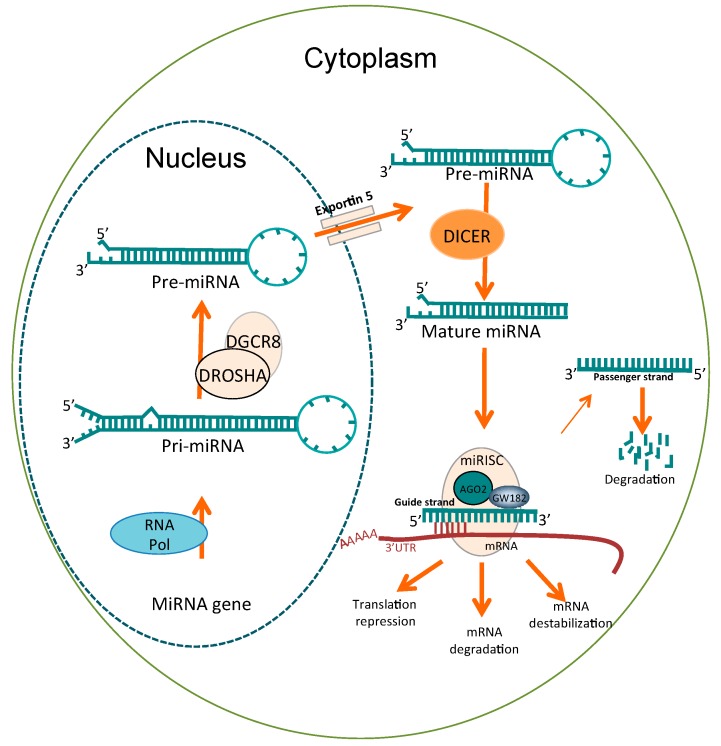
Biogenesis of miRNAs in the cell. miRNA precursors (pre-miRNAs) are transcribed in the nucleus and processed by the Drosha complex to generate pre-miRNAs. Pre-miRNAs are exported to the cytoplasm via exportin-5 and excised by DICER into a mature form of double-stranded RNA ~22 nt long. Double-stranded RNA is loaded onto AGO2 that is the catalytic component of the miRISC complex. One strand is removed from the duplex (the passenger strand) and the remaining RNA strand (the guide strand) binds to complementary sequences typically located in the 3’ untranslated region (UTR) of target mRNAs to repress translation or trigger mRNA cleavage.

The biogenesis of miRNAs (Figure 1) starts in the cell nucleus through the transcription of miRNA genes by RNA polymerase II (Pol II), which generates the primary miRNA precursors known as pri-miRNAs. Pri-miRNAs (60–100 nt) have a “hairpin” structure, consisting of a stem of 33–35 base pairs with a terminal loop [18,39]; pri-miRNAs are enzymatically cleaved by the Drosha-DGCR8 protein complex to produce a smaller precursor called pre-miRNA (~70 nt). These pre-miRNAs are transported to the cytoplasm through exportin-5-RanGTP. In the cytoplasm, pre-miRNAs are cleaved by DICER type III RNase, which produces mature ~22 nt miRNAs. Mature miRNAs are recruited by the protein complex called RISC or miRISC through its protein component Argonauta (AGO). The double strand of mature miRNA dissociates, and a single strand is retained (guide strand) in the miRISC complex. The guide strand recognizes the 3′ untranslated region (UTR) of its target mRNA, which has partial sequence complementary; however, this binding must exhibit perfect complementarity in a region of 2–8 nt called the seed region, which is located at the 5′ end of miRNAs [18,40]. The binding of miRNA to its target mRNA induces the blocking of translation by three possible mechanisms: translation repression, mRNA degradation and mRNA destabilization [41,42,43].

**Figure 2:**
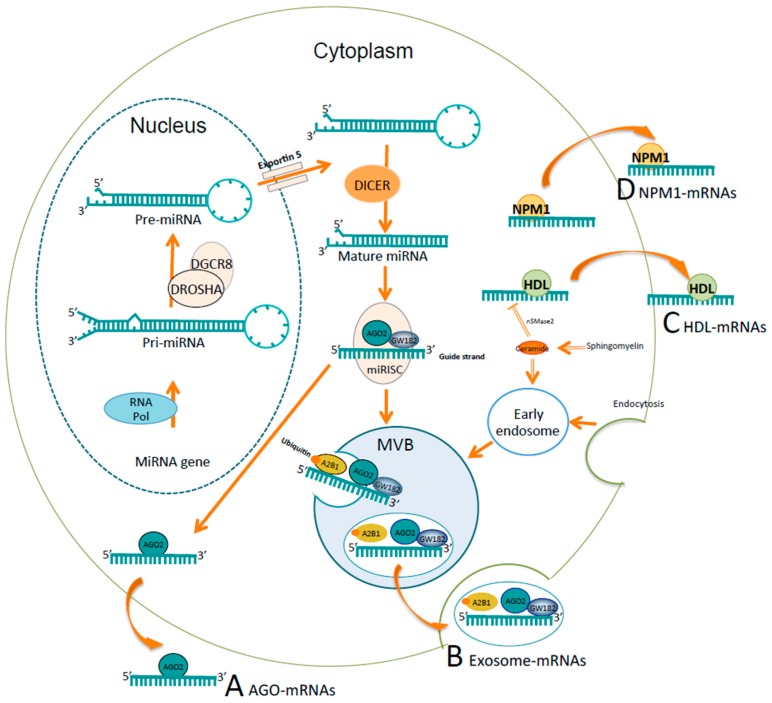
Mechanisms of secretion of miRNAs. (**A**) Secretion of the miRNAs associated with Argonaute2 protein (AGO2); (**B**) Secretion of the miRNAs by exosomes; (**C**) Secretion of the miRNAs associated with high-density lipoprotein (HDL); (**D**) Secretion of the miRNAs associated with the RNA binding protein nucleophosmin (NPM1). (**A**) miRNAs associated with AGO2, a main component of the RISC, can be stably exported into plasma samples. (**B**) miRNAs are sorted into multivesicular bodies (MVBs) derived from early endosomes. This mechanism requires ceramide production on the cytosolic side by neutral sphingomyelinase 2 (nSMase2), ESCRT machinery, and sumoylated hnRNPA2B1 protein, which specifically binds mature miRNAs and controls their loading into MVBs. MVBs are enriched with GW182 and AGO2, which are known to regulate the function of miRNAs. MVBs fuse with the plasma membrane and release exosomes into the extracellular medium. (**C**) miRNAs bound to HDL also can be stably exported into plasma samples via a mechanism repressed by nSMase2. (**D**) NPM1 binds miRNAs from the culture supernatants of tumor cell lines and fibroblasts while protecting them from RNase activity.

Regarding miRNAs in body fluids, four mechanisms of secretion of miRNAs have been described to date: (a) inside exosomes; (b) associated with AGO2 protein; (c) associated with high-density lipoprotein (HDL); and (d) associated with nucleophosmin 1 protein (NPM1) [36]. As shown in Figure 2, miRNAs are sorted into multivesicular bodies (MVBs) derived from early endosomes, which is a process that requires neutral sphingomyelinase 2 (nSMase2), endosomal sorting complex transport machinery (ESCRT) and sumoylated hnRNPA2B1 protein [44,45,46]. MVBs are enriched with two main components of the miRISC, GW182 and AGO2, which could be associated with miRNA functionality [36,47]. Finally, MVBs fuse with the plasma membrane and release exosomes into the extracellular medium. It has been shown that exosomes carrying miRNAs fuse with the plasma membrane of target cells and that the delivered miRNAs are functional inside the target cell [48,49]. In addition, miRNAs associated with Argonaute2 protein (AGO2) [50,51] and miRNAs bound to HDL [52,53] can be exported stably into human plasma samples. HLD-associated miRNAs are transferred actively to target cells in a functional form [52,54]; however, there is no experimental evidence for AGO-associated miRNAs; miRNAs released by these two mechanisms have not been reported in clinical samples from cancer patients. Lastly, one study reported that the RNA binding protein nucleophosmin (NPM1) binds miRNAs from the culture supernatants of tumor cell lines and fibroblasts while protecting them from RNase activity [55]. NPM1-associated miRNAs have not been reported in clinical samples or as part of active transfer into target cells.

## 3. Studies Regarding microRNAs as Biomarkers for MPM

A biomarker in cancer can be any cellular, molecular or genetic component that can be measured and associated with the neoplastic process or the presence of disease [56,57]. Ideally, a biomarker for cancer diagnosis should distinguish between patients with a specific type of cancer and those who do not have the disease with high specificity and sensitivity. It also should have high stability in biological samples and should be measured with a simple, accurate and reproducible method in any laboratory [56,57].

Most studies with the main goal to identify candidate miRNAs with diagnostic value started by identifying miRNAs differentially expressed in tumor samples compared to non-tumor samples, which is known as the discovery phase. During the discovery phase, high-throughput techniques, such as semi-quantitative microarrays and deep sequencing, enable the analysis of an extensive number of miRNAs, but they usually have a limited number of samples due to the high cost of these methods. Alternatively, a limited number of miRNAs are tested as potential biomarkers based on a hypothesis-driven method. The following validation phase is usually performed using a quantitative technique, such as reverse transcription quantitative real-time PCR (RT-qPCR), preferably on a larger number of samples obtained from an independent set of patients.

In addition to studies with a main goal to identify candidate miRNAs with diagnostic value, studies that aimed to identify miRNAs involved in the oncogenic mechanisms of MPM and those that analyzed miRNA levels in biological samples are addressed in this section. Certainly, the potential association of miRNAs with the MPM carcinogenic process increases the likelihood of miRNAs having diagnostic value for this disease.

Here, studies regarding miRNAs with potential diagnostic value for MPM are described with an emphasis on relevant aspects of the experimental strategy, which includes the following: (i) source, size and preservation method of the tumor samples and the samples that they are compared against (normal controls, healthy controls or another cancers); (ii) specific assays used for miRNA analysis in the discovery and validation phase; (iii) number of miRNAs analyzed; (iv) sensitivity and specificity values (Receiver operating characteristic curve analysis); and (v) potential limitations of the experimental design.

The studies are listed by the year of publication and sectioned by the type of sample source for clarity. Table 1 summarizes the described studies.

### 3.1. Studies in Pleural Tissue

Guled et al. [58] reported the first study aimed to evaluate the expression of miRNAs in MPM in 2009. The authors evaluated the expression of 723 miRNAs in 17 frozen MPM tissue samples and in commercially available total RNA from normal human pericardium as a control (Ambion, Austin, TX, USA) using microarrays (Agilent human miRNAs V2). They reported 12 over-expressed miRNAs (let-7b*, miR-1228*, miR-195*, miR-30b*, miR-32*, miR-345, miR-483-3p, miR-584, miR-595, miR-615-3p, and miR-885-3p) and nine sub-expressed (let-7e*, miR-144*, miR-203, miR-340*, miR-34a*, miR-423, miR-582, miR-7-1* and miR-9) in MPM tissue compared to the single control. The authors also compared miRNA expression between MPM samples and reported seven miRNAs that were expressed exclusively in the epithelioid subtype, five in the biphasic subtype and three in the sarcomatoid subtype. Bioinformatic analysis was used to identify three suppressor genes (CDKN2A, NF2 and RB1) as putative targets of over-expressed miR-30b*, miR-32*, miR-483-3p, miR-584, and miR-885-3p, whereas oncogenes hepatocyte growth factor (HGF), Platelet Derived Growth Factor Subunit (PDGFA), Epidermal Growth Factor (EGF) and Jun proto-oncogene (JUN) were identified as putative targets of sub-expressed miR-9, miR-7-1* and miR-203. Nevertheless, experimental assays were not performed to verify these findings. There are major limitations in this study; first, only one normal control (total RNA from one donor) was used in the discovery phase, and second, quantitative validation of the microarray results was not performed. There was no follow up work for this study.

In 2010, Gee et al. [59], in order to identify biomarkers for the differential diagnosis of MPM and lung adenocarcinoma (AD), analyzed miRNA expression profiles of pleural tissue samples from MPM patients (*n* = 15) and AD patients (*n* = 10) using microarrays (Affymetrix). Microarray data were validated by RT-qPCR in 100 samples of MPM and 32 samples of AD. The authors reported seven miRNAs sub-expressed in MPM that could discriminate MPM from AD with greater than 80% specificity and sensitivity (miR-200c, miR-200b, miR-141, miR-429, miR-203 and miR-205), providing the first evidence of potential differential diagnosis biomarkers for these different tumors that are often difficult to differentiate histologically.

Also in 2010, Benjamin et al. [60] performed a study to identify biomarkers that could distinguish MPM from other carcinomas of epithelial origin that can invade pleura. In the discovery phase, seven pleural tissue samples of MPM and 97 tissue samples of epithelial carcinomas from various organs (lung, bladder, breast, and kidney, among others) were analyzed by microarray (Nexterion Slide E, Schott, Mainz, Germany). The results indicated that miR-193a-3p was over-expressed in MPM vs. all carcinomas, miR-200c was sub-expressed in MPM vs. renal cell carcinoma (RCC) and miR-192 was sub-expressed in MPM vs. non-RCC carcinomas. It should be noted that miR-200c was also reported by Gee et al. [59] as down-regulated in MPM compared to AD. These results were validated in 22 MPM and 43 carcinoma samples (new samples added: 15 MPM and 36 carcinomas) by RT-qPCR. The sensitivity and specificity of using these three miRNAs to diagnose MPM were analyzed in 32 MPM and 113 carcinoma samples (new samples added: 11 MPM and 77 carcinomas), resulting in a sensitivity of 97% and a specificity of 96%. Finally, a blind study of 63 new samples of pleural and lung tissue (14 MPM and 49 metastatic carcinomas) was performed. The results indicated that 14 MPM samples were correctly identified (100% sensitivity) and that 46 of the 49 carcinomas were correctly identified (94% specificity). This report was the first study that provided a quantitative diagnostic tool (RT-qPCR) to discriminate MPM vs. other epithelial carcinomas that may invade pleura and MPM vs. lung adenocarcinoma.

In 2011, Santarelli et al. [29] analyzed miRNA expression profiles in the pleural tissue of MPM patients and matched adjacent non-neoplastic pleural samples to identify candidate biomarkers for the diagnosis of MPM. They quantified the levels of 88 miRNAs that were previously reported to be associated with cancerous processes in ten samples of MPM and one sample of healthy mesothelial tissue from an RNA-pool of five individuals using a customized PCRArray (Array MAH-102A, SABiosciences). These samples were previously preserved in RNALater solution (Ambion) at −80 °C. Three sub-expressed miRNAs in MPM that were identified in PCRArray analysis (miR-335, miR-126 and miR-32) were subsequently analyzed by RT-qPCR in 27 formalin-fixed paraffin-embedded (FFPE) samples of MPM and 27 adjacent healthy pleural tissues. The data indicated that only hsa-miR-126 showed significant sub-expression in MPM compared to healthy tissues. It should be noted that this study was limited to the analysis of 88 microRNAs in the discovery phase and that samples were preserved with different methods. It has been reported that some miRNAs detected in frozen tissue samples may vary from those detected in FFPE samples because of the degradation of some miRNAs in the latter; however, these profiles were shown to be comparable [61]. Samples preserved in FFPE are frequently more available than frozen samples; therefore, this information is valuable for future studies regarding diagnostic purposes. Nevertheless, there were no follow up studies in malignant tissues from these authors.

In 2013, Xu et al. [62] performed miRNA profiling to identify altered miRNAs in MPM tissues that could be associated with the oncogenic transformation of mesothelial cells. They analyzed miRNA expression in 25 MPM specimens and six normal parietal pleural samples from patients without mesothelioma or other malignancies by microarray (BeadChips v2, Illumina). The results indicated that 49 miRNAs were over-expressed and 65 were under-expressed in MPM compared to controls. The authors reported the validation of four miRNAs by RT-qPCR (sub-expressed miR-206, miR-1 and miR-483-5p; over-expressed miR-155*), but they did not state clearly whether a new group of tissue samples was used for this validation. Because this study aimed to identify biologically relevant miRNAs in the development of MPM instead of miRNAs with diagnostic value, under-expressed miR-1 was transfected into two MPM cell lines (H513, epithelioid type and H2052, sarcomatoid type), which caused cycle arrest and apoptosis. This effect was associated with increased mRNA expression of the tumor suppressors p53, Bcl2-associated X protein (BAX) and cyclin-dependent kinase inhibitors p16/p21 and decreased mRNA expression of anti-apoptotic Bcl-2 and surviving. However, direct experimental evidence of this association was not provided in the paper. None of the putative target genes of the altered miRNAs identified by informatics analysis were tested experimentally.

Reid et al. [63] in 2013 reported that miR-16, miR15a, miR-15b, and miR-195 were markedly sub-expressed in FFPE tissue samples of MPM patients (*n* = 60) compared to normal pleural tissue samples of cardiac surgery patients (*n* = 23). Four-fold to 22-fold (miR-16) under-expression was verified in four MPM cell lines relative to the normal mesothelial cell line MeT-5A (two-fold to five-fold). The expression of miR16/15 was restored in cell lines to elucidate their biological function in MPM to find a new potential target for therapy. Restoring miR-16 using synthetic mimics resulted in growth inhibition, cell cycle arrest in G0/G1, increased apoptosis and reduced colony formation in the MPM cell line MSTO-211H but not in MeT-5A cells. These effects correlated with down-regulation of the miR-16 known target anti-apoptotic gene Bcl-2 and cyclin D1-encoding gene (CCND1) in H28 and MSTO-211H cell lines. Further, intravenous injection of miR-16-containing minicells to nude mice already xenografted with MSTO-211H cells led to tumor growth inhibition that was dose-dependent.

In 2014, Cioce et al. [64] performed a screening test (887 miRNAs) on 29 pleural tissue samples of MPM vs. 12 tissue samples of peritoneal mesothelial cysts (preserved in FFPE) with microarrays (Human miRNA Microarray V2, Agilent). Among the 19 miRNAs differentially expressed in MPM, sub-expressed miR-145 was chosen for validation by RT-qPCR in fresh pleural tissue samples of MPM (*n* = 6) and normal tissue (*n* = 14) and subsequently in frozen samples of pleural tissue with MPM (*n* = 36) and normal peritoneal mesothelial tissue (*n* = 36, same patients). They proceeded with over-expression of miR-145 in three MPM cell lines and a normal mesothelial cell line and reported reductions in proliferation and migration in two of the MPM cell lines compared to the control. They further performed a xenotransplant of MSTO-211H cells that over-expressed miR-145 in SCID mice and observed an inhibition of tumor growth in six of eight treated mice compared to controls. The authors concluded that the results suggested that miR-145 functions as a tumor suppressor; however, the number of experimental animals and cell lines were rather limited to provide reliable evidence. In addition, peritoneal mesothelial cyst tissue was used as a comparative control group in the discovery phase. Cystic mesothelial lesions are benign; therefore, they may not be comparable to the validation phase when normal peritoneal mesothelial tissue was used as a control. Like the study reported by Xu [62], this paper does not focus on the diagnostic value of miR-145 in MPM but on its potential association with a carcinogenic process. Additionally, this study also reported the sub-expression of miR-200c, which coincides with the results reported by Gee, et al. in 2011 [59].

Ramirez-Salazar et al. [65] in 2014 analyzed miRNA expression profiles in pleural tissue with epithelioid MPM (*n* = 5), pachypleuritis (PP) (*n* = 4) and atypical mesothelial hyperplasia (HP) (*n* = 5) and in non-cancerous/non-inflammatory tissue (*n* = 5) as a control using PCRArray (TaqMan Array, Applied Biosystems). The aim of this study was to elucidate mechanisms associated with the development of MPM since pleural chronic inflammation is considered to be a detonating factor in MPM pathogenesis. Different from most studies on mesothelioma, this study provided a description of the histological diagnosis of all tissue samples. Moreover, only tumor samples containing >80% neoplastic cells were used, which provided better tumor representativeness. The authors reported 19 miRNAs that were differentially expressed in MPM samples compared to control samples, 11 that were sub-expressed (miR-517b-3p, miR-627, miR-766-3p, miR-101-3p, miR-501-3p, miR-212-3p, miR-596, miR-145-5p, miR-671-3p, miR-181a-5p and miR-18a-3p), and eight that were over-expressed (miR-30e-3p, miR-34a-3p, miR-622, let-7a-5p, miR-196b-5p, miR-135b-5p, miR-18a-5p and miR-302-3p). Bioinformatic analysis revealed that the targets of four under-expressed miRNAs in MPM (miR-181a-5p, miR-101-3p, miR-145-5p and miR-212-3p), one in PP (miR-101-3p) and one in HP (miR-494) were significantly associated with “cancer pathways”. Nevertheless, the authors did not perform any experimental studies to assess the predictive results. Coincidently, Cioce et al. also reported the sub-expression of miR-145 in MPM [64].

Andersen et al. [66] sought to identify miRNA candidates for diagnostic biomarkers by analyzing miRNA expression in samples preserved in FFPE of five MPM specimens previously treated with chemotherapy (MPM), five preoperative diagnostic biopsy samples of MPM (DB) and five non-neoplastic pleural tissue samples of a patient with MPM previously treated with chemotherapy (NNP) using PCRArray (miRCURYLNA Universal RT microRNA Ready-to-Use, Human Panels I + II v2). The authors chose four sub-expressed (miR-126, miR-143, miR-145 and miR-653) and two over-expressed (miR-193a-3p, miR-193b) miRNAs found in either the DB or MPM samples compared to NNP samples for RT-qPCR validation using 40 MPM, 12 DB and 14 NNP samples. The results indicated statistically significant sub-expression of miR-126, miR-652, miR-145 and miR-143 in both DB and MMP compared to NNP. It was reported that miR-145 and miR-652 had a sensitivity or specificity >80%, whereas miR-143 and miR-126 had a sensitivity or specificity <80% (Receiver operating characteristic curve or ROC curve). It is important to note the main potential design limitations of this study: first, the samples analyzed at the screening phase contained 40–85% neoplastic tissue, but it was not clear how many samples contained specific percentages in that range. This point could be relevant if we consider the representativeness of each sample as a tumor whose non-neoplastic content was 60% versus 15%. Second, the authors stated that in order to test for any chemotherapy-induced changes in miRNA expression profiles, they had to compare diagnostic biopsy samples without treatment (DB) to NNP; however, the latter samples were previously treated with chemotherapy. Chemotherapy affects normal and tumor tissues, potentially inducing changes in the miRNA expression profiles of both; therefore, this aim cannot be achieved with the stated comparison. Nevertheless, this study was the third to report the sub-expression of miR-145 in MPM pleural tissue.

In 2015, Ak et al. [67] investigated miRNA expression levels in frozen pleural tissues from MPM and benign asbestos-related pleural effusion (BAPE) patients using PCRArray. BAPE tissue samples showed non-specific pleuritis/fibrosis. The authors performed PCRArray (384 miRNAs, Applied Biosystems) on 18 MPM (11 with chemotherapy treatment) and six BAPE samples and reported 11 over-expressed miRNAs in MPM samples compared to BAPE (miR-484, miR-320, let-7a, miR-744, miR-20a, miR-193b, let-7d, miR-125a-5p, miR-92a, miR-155, and miR-152). They evaluated the diagnostic value of these miRNAs to differentiate MPM from BAPE using ROC and area under the curve (AUC) analysis. The results showed that four miRNAs had AUC values ≥0.90 (miR-484, miR-320, let-7a and miR-125a-5p). Meanwhile, miR-484 had a sensitivity and specificity of 100%, miR-320 had a sensitivity of 78% and a specificity of 100%, let-7a had a sensitivity of 94% and a specificity of 83% and miR-125a-5p had a sensitivity of 89% and a specificity of 100%. This study had some limitations: using a mixture of tissue samples from MPM patients treated with chemotherapy and without treatment and a limited number of samples without validation in an independent cohort of patients. Potential chemotherapy-induced changes in the miRNA expression profiles may have induced bias in the analysis.

Also in 2015, Birnie et al. [68] investigated the role of miR-223 in MPM based on evidence that suggested that miR-223 might be a tumor suppressor in other hematopoietic and solid tumors and on their own initial findings that indicated that miR-223 was down-regulated in three MPM cell lines compared to one human mesothelial cell control. They examined the expression levels of miR-223 in 17 MPM pleural tissue samples and six normal pleural tissue samples from non-cancer patients undergoing cardiac or aortic surgery by RT-qPCR. They enriched the tumor content of the FFPE-conserved specimens by performing laser-capture micro-dissection. In addition, they examined miR-223 expression in cells obtained from the pleural effusion of 26 MPM and ten benign pleural disease patients. Down-regulation of miR-223 was confirmed in MPM tissues and MPM effusion cells. After over-expression of miR-223 in MPM cell lines (Human JU77 and CRL2081), STMN1 levels were reduced, cell motility was inhibited, and tubulin acetylation was induced. Migration of both cell lines was significantly reduced following the knockdown of STMN1 expression. In addition, miR-223 levels increased, whereas STMN1 was reduced following the re-expression of c-Jun N-terminal kinase (JNK) isoforms in JNK-null murine embryonic fibroblasts, suggesting that miR-223 and its target STMN1 are involved in the regulation of MPM cell motility, which may be associated with their carcinogenic properties.

In 2016, Cappellesso et al. [69] searched for miRNAs that could be used as a complementary tool for the diagnosis of MPM in pleural effusion cytology. For this study, the authors decided to test 15 miRNAs previously reported by three publications as potential candidates for MPM biomarkers using RT-qPCR. First, they analyzed miRNA expression levels in two MPM cell lines (H2052 and H28) and one normal mesothelium cell line (MET-5A) and reported the over-expression of miR-19a, miR-19b, miR-21 and miR-25 and sub-expression of miR-126. These miRNAs were further analyzed in pleural tissue samples preserved in FFPE of 51 MPM and 40 benign/reactive pleurae with the same results. Likewise, these five miRNAs were evaluated in 29 cytological samples of MPM and 24 cytological samples with reactive mesothelial cells (RMCs). It was indicated that 31 samples were air-dried and stained with May–Grunwald–Giemsa and 22 samples were fixed in 95% alcohol and stained with Papanicolaou, but the authors did not clarify which type of samples were stained (MPM or RMCs). They found over-expression of miR-19a and miR-21 and sub-expression of miR-126 in MPM compared to RMCs. ROC analysis suggested that miR-19a, miR-19b, miR-21 and miR-126 could be diagnostic biomarkers of MPM in cytological samples because they showed a sensitivity or specificity >0.80. The results showed that the five analyzed miRNAs were detectable in these samples. One striking detail is that this study is the first to report the quantification of miRNAs from stained cytological samples. The authors stated that “staining, fixation, and the length of time in storage did not markedly affect final RNA quality or yield among smears”, but they did not provide evidence to support this statement. It would have been relevant to this field and to other researchers to report this evidence as new findings.

In a subsequent study, Cappellesso et al. (2017) [70] searched for candidate biomarker miRNAs for differential diagnosis of MPM from AD in histologic and cytological specimens. First, a bioinformatic analysis of three data sets regarding the expression of miRNAs in MPM and AD was performed to select candidate miRNAs. Three upregulated miRNAs (miR-130a, miR-193a, and miR-675) and three downregulated miRNAs (miR-141, miR-205, and miR-375) in MPM vs. AD were selected. Their expression was tested in 41 epithelioid MPM and 40 AD histologic specimens (FFPE) and 26 MPM and 27 AD cytological specimens, by RT-qPCR. In this study, tumor cells were microdissected manually from histologic samples and the cells were scraped from each slide from cytological samples to ensure a tumor cell content >70%. Results indicated that only miR-130a was significantly overexpressed in both histologic and cytological MPM specimens compared to AD. Finally, the ROC analysis of miR-130a in cytological samples demonstrated a low sensitivity of 77%, a specificity of 67%.

### 3.2. Studies in the Cellular Fraction of Peripheral Blood

Tissue miRNAs have promising diagnostic value for several tumors, including MPM. However, obtaining pleural tissue samples requires invasive procedures. An alternative is using biological samples than can be obtained with less invasive procedures, such as peripheral blood.

In 2012, Weber et al. [71] published the first study that analyzed the diagnostic value of miRNAs in the cell fraction of human peripheral blood of MPM patients, cancer-free asbestos-exposed individuals (AEC) and cancer-free individuals from the general population (CGP). They first analyzed 328 miRNAs in 23 MPM and 17 AEC using microarrays (miRVana miRNA Probe Set v3.3, Ambion, TX, USA) and found that miR-20a and miR-103 were under-expressed in MPM. These two miRNAs were quantified in 23 MPM, 17 AEC and 25 CGP by RT-qPCR, and the results showed that only miR-103 was significantly under-expressed in MPM. ROC analysis showed that miR-103 could discriminate MPM from AEC with a sensitivity of 83% and specificity of 71% and could discriminate MPM from CGP with a sensitivity of 78% and specificity of 76%.

In 2014, Weber et al. published a follow up paper [72] in which they analyzed the diagnostic value of a combination of miR-103a-3p levels in the cell fraction of peripheral blood (by RT-qPCR) and the mesothelin concentration in plasma (ELISA test). The analysis of 43 male MPM patients and 52 male controls formerly exposed to asbestos revealed that the combination of mesothelin and miR-103a-3p showed a sensitivity of 95% and a specificity of 81% for MPM diagnosis. For individual determinations, mesothelin showed a sensitivity of 74% and specificity of 85%, whereas miR-103a-3p showed a sensitivity of 89% and a specificity of 63%.

These two studies did not provide details on how the healthy status of normal controls and asbestos-exposed subjects were assessed.

### 3.3. Studies in Serum and Plasma

MicroRNAs are secreted into the liquid fraction of peripheral blood (serum and plasma) and into all body fluids analyzed to date. These studies described here focus on miRNAs found in serum and plasma (also known as circulating miRNAs) as potential biomarkers for MPM.

In their study performed in 2011, Santarelli et al. [29] also evaluated the levels of miR-126 in serum samples of 44 MPM patients, 196 asbestos-exposed subjects and 50 healthy subjects together with the levels of soluble mesothelin-related peptides (SMRPs) using RT-qPCR and ELISA, respectively. ROC analysis showed that cut-off values of miR-126 discriminated asbestos-exposed subjects from controls with a sensitivity of 60% and specificity of 74%, and from MPM patients with a sensitivity of 73% and specificity of 74%. These values are promising, although the recommended values for good biomarkers are a sensitivity and specificity >80%. One advantage is the large number of samples analyzed. In addition, asbestos-exposed subjects were very well classified as a control group in this study. Chest radiography and high-resolution computed tomography were performed to verify the absence of tumors, and detailed questionnaires on asbestos exposure were administered. The authors also reported that a combination of decreased levels of miR-126 and increased levels of SMRPs correlated with a higher risk of developing MPM, but they did not report sensitivity or specificity values for that determination.

In a subsequent study (2012), Tomasetti et al. [73] aimed to validate an optimized method for the detection of miR-126 in serum. In this new contribution, endogenous and exogenous controls were used for the normalization of RT-qPCR data, the accuracy and precision of the method were evaluated, and relative plus absolute RT-qPCR quantification was performed. The authors quantified miR-126 in diluted serum samples of 45 MPM, 20 non-small cells lung cancer (NSCLC) patients and 56 healthy controls using RT-qPCR. The results showed an under-expression of miR-126 in MPM and NSCLC compared to healthy controls, which significantly discriminated MPM patients from healthy controls and from NSCLC patients, but did not differentiate NSCLC patients from healthy controls. ROC analysis indicated that miR-126 in serum is a candidate biomarker for MPM with high sensitivity (80%) but low specificity (60%).

Also in 2012, Kirschner et al. [74] analyzed miRNA expression profiles in the plasma of five MPM patients (three epithelioid and two sarcomatoid types) and three healthy controls using microarrays (V3, miRBase V12.0, Agilent Technologies, Santa Clara, CA, USA). They found 15 over-expressed miRNAs in MPM compared to controls. The authors further validated the microarray results of 12 candidate miRNAs with the most significant elevation levels in the plasma of 15 MPM patients and 14 control subjects (eight patients with coronary artery disease and six healthy subjects) using RT-qPCR. The results indicated that only miR-625-3p was significantly over-expressed in MPM. ROC analysis showed that plasma miR-625-3p levels discriminated between MPM patients and control subjects with an accuracy of 82.4%, a sensitivity of 73.3% and a specificity of 78.5%. Instead of testing a larger cohort of plasma samples, the levels of miR-625-3p were quantified in serum from a new cohort of 30 MPM patients and ten subjects with asbestosis. Levels of miR-625-3p were significantly elevated in MPM patients compared with asbestosis patients with an accuracy of 79.3%, a sensitivity of 70% and a specificity of 90%. Then, the authors decided to evaluate the levels of 12 miRNAs identified in microarray analysis of 18 tissue samples from MPM patients and seven pericardial tissue samples used as controls. Unlike previous results in plasma and serum, the data indicated an over-expression of miR-625-3p and an under-expression of miR-29c*, miR-16, miR-196b, miR-26a-2-3p and miR-1914-3p in MPM tumor samples compared to controls. These mismatched data are not surprising; there are studies that suggest that miRNA expression profiles in cells/tissues do not necessarily reflect the profiles of their secreted miRNAs [75,76]. The disadvantages of this study include a limited number of samples for each type of biological sample used. Perhaps choosing a larger number of plasma samples instead of analyzing a very limited number of serum and tissue samples would have been more useful. In addition, the authors used plasma from eight patients with coronary artery disease (together with six healthy subjects) as normal controls in the validation phase. These samples should not be considered controls because they are not from healthy subjects and because miRNA profiles can be altered due to coronary artery conditions [77,78]. Details on how the healthy status of control subjects was assessed were not provided. Later, levels of miR-16 were tested in a larger number of tissue samples (60 MPM samples) in a paper published in 2013 [63], but they were not tested in serum or plasma samples.

On the other hand, Gayosso-Gómez (2014) [79] searched for candidate biomarker miRNAs for differential diagnosis of MPM from AD in serum. They analyzed the miRNA profiles of pooled serum samples of 22 MPM (epithelioid), 36 AD patients and 45 healthy controls using deep sequencing (Illumina). The results indicated over-expression of seven miRNAs in MPM and 12 miRNAs in AD patients compared to healthy controls. Among these miRNAs, four were common to both neoplasms (miR-4791, miR-185-5p, miR-96-5p and miR-1271-5p), whereas miR-1292, miR-409-5 and miR-92b -5p were over-expressed exclusively in MPM. Comparative analysis of MPM vs. AD patients showed 13 miRNAs over-expressed and five miRNAs sub-expressed in MPM patients. The disadvantages of this study are the lack of quantitative validation of the sequencing results and the lack of a follow up study. An advantage was that the respiratory function of healthy subjects was verified.

In 2015, Lamberti et al. [80] reported the identification of two serum miRNA signatures that correlate with the clinical outcome and histological subtype of MPM. They quantified 384 miRNAs in the serum of 14 MPM patients (seven epithelioid, three sarcomatoid and four biphasic types) and ten patients affected by non-cancer-related pleural effusions as normal controls using PCRArray (Microfluidic card A, Applied Biosystems). The results indicated over-expression of miR-101, miR-25, miR-26b, miR-335 and miR-433 and sub-expression of miR-191 and miR-223 in MPM. Additionally, miR-29a and miR-516 were detected exclusively in MPM patients. Notably, it was stated that RT-qPCR was performed to evaluate miRNAs in “an extended group of patients”, but clear information about these patients was not provided in the study. Patients were subdivided into two groups: group A, which was composed of patients with over-expression of ≥3/9 miRNAs and miR-516 undetectable or unchanged, and group B, which contained patients with at least 3/9 miRNAs sub-expressed or without change and/or miR-29a sub-expressed. Based on these criteria, patients in group A (*n* = 5) had a significantly shorter mean survival than patients in group B (*n* = 9) (7 months vs. 17 months, *p* = 0.0021). They reported that in patients with signature A, 2/5 had sarcomatoid and 3/5 had biphasic MPM, but statistical significance was not provided in this study. Therefore, two important pieces of information are missing. In addition, MPM patients were compared to non-cancer related pleural effusion patients as normal controls. Pleural effusion patients should not be considered normal or healthy controls. Pleural effusion does not occur in healthy subjects. In addition to tumor-related conditions, etiologies of pleural effusions are diverse and range from cardiopulmonary disorders to systemic inflammatory conditions that could be infectious (viral or bacterial) or non-infectious. More importantly, the authors did not report the diagnosis or etiology of the ten pleural effusion patients; therefore, there is no information on a potential bias in case of different etiologies. In addition to differences in pathogenesis and clinical characteristics, for example, viral or bacterial pneumonitis vs. cardiac failure, it has been suggested that miRNA expression profiles are distinctive for different diseases [81,82,83,84]. Perhaps the initial approach to this study should have considered the discovery of miRNAs in MPM patients that can distinguish them from patients with other non-cancer related diseases that induce pleural effusion, which also would have clinical value.

In a new contribution, Santarelli et al. [85] analyzed the diagnostic value of a combination of three markers (miR-126, methylated thrombomodulin promoter or Met-TM and SMRPs) in serum samples of 45 MPM patients, 99 asbestos-exposed subjects and 44 healthy controls to detect MPM. They further evaluated the three biomarkers in 18 MPM, 50 asbestos-exposed subjects, 20 healthy controls and 42 lung cancer (LC) patients. The population of LC patients was included for cancer specificity evaluation. The data indicated that the risk of MPM significantly increased at high SMRP levels with at least one or both altered epigenetic biomarkers (low miR-16 or high Met-TM), whereas the disease risk was maximum when all three biomarkers were altered. Conversely, the LC patients showed low miR-126 and high Met-TM levels but were associated with low levels of SMRPs. The combination of SMRPs, miR-126 and Met-TM improves the differential diagnosis of MPM up to an AUC of 0.857 (95% CI, 0.767–0.927) compared to SMRP alone at 0.818 (95% CI, 0.723–0.914). Importantly, the authors reported that the expression of these biomarkers was independent of gender, age, smoking and duration of asbestos exposure, which is a new contribution for MPM-related studies.

In 2016, Bononi et al. [86] investigated miRNA expression profiles in the serum of ten MPM patients, ten subjects exposed to asbestos (AE) and ten healthy subjects (HC) by using microarrays (Agilent Technologies, Human miRNA G4470A) in the discovery phase. Out of 37 differentially expressed miRNAs in MPM, three were validated in 30 sera previously used for microarray analysis and in additional 19 serum samples (ten MPM, five AE and four HC) by RT-qPCR. The results indicated that miR-197-3p, miR-1281 and miR-32-3p were up-regulated in MPM compared to HC; miR-197-3p and miR-32-3p were up-regulated in MPM compared to AE; and miR-1281 was up-regulated in MPM and AE compared to HC. AUC in all cases were a little less than 0.8. This work was one of few studies that identified endogenous stable miRNA that could be used as suitable normalizer. On the other hand, some relevant information was missing, such as the histological subtypes of MPM patients and how the healthy status of the controls was verified. This study did not find the down-regulation of miR-126 in MPM as previously reported by Santarelli et al. [29,85], who also used serum from asbestos-exposed subjects as comparative controls.

Cavalleri et al. [87] in 2017 aimed to identify a specific miRNA signature in plasmatic extracellular vesicles (EV) that discriminates MPM patients from past asbestos-exposed subjects (PAE). They analyzed 754 miRNAs in plasmatic EVs of 23 MPM patients and 19 cancer-free subjects exposed to asbestos in the past using an OpenArray method. Among 62 miRNAs differentially expressed in MPM compared to PAE (sub-expression), 16 out of 20 analyzed miRNAs were quantitatively confirmed by RT-qPCR. The authors found a signature of the five best discriminating miRNAs of miR-103a-3p, miR-98, miR-148b, miR-744 and miR-30e-3p with an AUC of 0.864, 0.864, 0.852, 0.845 and 0.827, respectively. They further simplified the signature with miR-30e-3p and miR-103a-3p, which generated an AUC of 0.942 with a sensitivity of 95.5% and specificity of 80%. Down-regulation of miR-103a-3p was also found in the cellular fraction of peripheral blood of MPM patients in two previous reports [71,72]. In addition, the authors tested other miRNAs reported in the literature as potential biomarkers of MPM (miR-126, miR-625-3p, miR-25, miR-29 and miR-433) and did not find significant differences between the study groups. Nevertheless, the evidence indicates that the type and levels of miRNAs may vary in the exosomal fraction compared to those found in exosome-free fraction and may vary between different body fluids, such as serum vs. plasma [36]. This work is the first study that exclusively analyzed the miRNAs in exosomes isolated from plasma samples of MPM patients, which may provide relevant information about miRNA release mechanisms associated with neoplastic processes for future studies. However, a potential disadvantage of this approach for future clinical applications is that ultracentrifugation requires a specialized heavy apparatus (ultracentrifuge) that is not common in clinical laboratories and two extra hours of sample processing.

In 2017, Weber et al. [88] aimed to identify candidate biomarker miRNAs in plasma for diagnosis of MPM. For the discovery phase, they analyzed 377 miRNAs in plasma of 21 MPM patients and 21 asbestos-exposed controls, using PCRArray (TaqMan Low density Array Human MicroRNA CardA v2.0). The authors reported the identification of three stable reference miRNAs (miR-20b, miR-28-3p and miR-146b-5p), and also reported that they normalized the raw *C*t values from the PCRArray with combinations of the three reference miRNAs to identify the candidate miRNAs. Then, miR-24 (miR-146b-5p as reference) and miR-132-3p (miR-146b-5p as reference), miR-24 (miR-146b-5pMiR-28-3p as reference) and miR-132-3p (miR-28-3p as reference) showed statistically significant down-regulation in MPM, and they were analyzed in the subsequent validation phase (22 MPM patients and 44 asbestos-exposed controls) using RT-qPCR. Results indicated that only miR-132-3p (and miR-146b-5p as reference) reached a significant difference in MPM patients compared to controls.

The authors additionally measured the expression of miR-126 (using U6 snoRNA as reference) and miR-625-3p (miR-16 as reference) in the verification phase to evaluate the discrimination potential of biomarker combinations. These miRNAs were previously described as candidate MPM biomarkers by Santarelli [29] and Kirschner et al. [74]. Results indicated that miR-126 was statistically significantly downregulated in MPM compared to controls. However, there are confusing details in this experimental design and results. First, miR-126 is included in the Human MicroRNA Card A v2.0 that was used for this study (discovery phase), and the authors did not report that the miR-126 expression was altered in MPM compared to controls. Therefore, it is not clear how the levels of miR-126 were found downregulated in the validation phase but not in the discovery phase in this study. Second, Santarelli et al., 2011 [29] used U6 snoRNA as a normalizer, but U6 snoRNA was not discovered as a suitable normalizer in this study, but it was used as reference anyway. Perhaps this inconsistency in miR-126 levels can be explained by the use of inadequate normalizer for these samples. Finally, the authors also reported that the combination of miR-132-3p and miR-126 within a panel (with two different references for normalization) implicate a less robust diagnosis method.

### 3.4. Studies in Other Body Fluids

To date, there are no publications that analyze the diagnostic value of secreted miRNAs in other biological fluids in MPM. Samples such as pleural effusion fluid could be a good option. This condition is a common clinical manifestation of late MPM, which is when most patients seek medical attention. Moreover, recent evidence suggests that secreted miRNAs in pleural effusion may have diagnostic value in other neoplasms, such as lung cancer [89]. As mentioned before, Birnie et al. analyzed the miRNA expression patterns of cells from pleural effusion [68]. Although they were not secreted miRNAs, miRNAs from pleural effusion cells would be a good option for potential non-invasive biomarkers.

## 4. Relevant Aspects of the Experimental Designs That May Influence the Accuracy, Consistency and Real Diagnostic Value of Currently Reported Data

### 4.1. Number of Samples

Most of the MPM-related studies analyzed a limited number of biological samples. Even for the validation phase, less than 100 samples were analyzed with a few exceptions. In fact, power and sample size calculations were not presented in any of the published studies. There is no doubt that data obtained from large-scale studies are considered the most reliable; however, it is relevant to notice that MPM is a low frequency disease that is difficult to diagnose and therefore samples may not be available in great number to the investigators at the time of research. For this particularly rare but aggressive tumor, information provided by well-designed studies will be relevant even with a limited number of samples. One strategy to overcome this scenario is to validate findings in follow up studies with larger, independent sets of patients when samples are available.

### 4.2. Follow up Studies of Promising Candidate miRNAs Biomarkers

Unfortunately, most of the studies reported data that were never confirmed in subsequent independent analysis with a few exceptions. Down-regulation of miR-126 was assessed in the serum of MPM patients in three subsequent studies by Santarelli et al. in 2011 [29], Tomasetti et al. in 2012 [73] and Santarelli et al. in 2015 [85]. Weber et al. reported the sub-expression of miR-103 in the cellular fraction of peripheral blood of MPM patients in two subsequent studies [71,72].

On the other hand, independent studies identified some common miRNAs as candidate biomarkers for MPM: in addition to Weber et al., an independent research group reported the sub-expression of miR-103 in the plasma of MPM patients [87]. Three independent authors found that miR-145 was downregulated in the pleural tissue of MPM patients [64,65,66].

Consistently reported miRNAs in different publications are more likely to have clinical relevance; therefore, a simple vote-counting method could be applied to choose promising candidate miRNAs that will be evaluated in statistically well-powered prospective studies. Table 2 summarizes the candidate miRNAs biomarkers of MPM reported by at least two independent studies and includes only RT-qPCR-validated miRNAs.

On the other hand, a meta-analysis is a better approach because it provides statistical analysis of multiple data, increasing the likelihood of finding good candidate miRNAs. However, performing a meta-analysis requires an investigator to decide how to search for studies, how to select those studies, which criteria to use and which data to include. These choices affect the results of this analysis.

Therefore, both vote-counting methods and meta-analysis should be performed carefully by choosing reliable data for the analysis, for example only RT-qPCR-validated miRNAs.

In this regard, Micolucci et al. (2016) [90] performed a systematic review and meta-analysis in order to identify high-confidence miRNAs that could serve as biomarkers of MPM compared to asbestos exposure subjects. First, they listed the most frequently reported miRNAs that had been described in 2–5 papers in their table 1 and supplementary table 1 by using a vote-counting method. Nevertheless, the authors listed all miRNAs reported in those papers without analyzing the reliability of the studies. For example, both tables included miRNAs that were identified by a single microarray analysis, without verification with RT-qPCR. Moreover, Table 1 included miR-20a (reported by Weber 2012 [71]), which was not significantly down-regulated in MPM after the verification test performed by Weber et al. themselves. Another example is miR-101, which was reported by Kemp et al. [91] as downregulated in only six tumor samples vs. three normal pleural tissues. Next, the authors conducted a qualitative meta-analysis using only RT-qPCR-validated miRNAs to improve the results of the vote-counting method, but details of other criteria were not provided. This method identified nine miRNAs as the most significant in tissue (miR-145-5p, miR-126-3p, miR-16-5p, miR-192-5p, miR-193a-3p, miR-200b-3p, miR-203-3p, miR-143-3p and miR-652-3p) and three circulating miRNAs (miR126-3p, miR-103a-3p and miR-625-3p). In addition, authors analyzed the biological function of these promising miRNAs to estimate their potential value as biomarkers. In spite of the heterogeneity of MPM studies, these qualitative meta-analyses and functional research provide an undeniably useful list of candidate miRNAs that should be analyzed in a large-scale study in order to assess their clinical relevance.

### 4.3. Relevance of Tumor Representativeness in Tissue Samples

It is notable that only two studies assessed tumor representativeness in the analyzed tissue samples by using only MPM samples with >80% tumor content and by using laser micro-dissection [65,68]. Few other studies mentioned the percentage of tumor content in MPM tissue samples, which ranged from 40–85% [58,60,62,66]. As previously mentioned, this information could be relevant if we consider the representative tumor content in a sample with a non-neoplastic content of 60% vs. 15%, for example. Remember that a potential diagnostic value is based on the hypothesis that tumor tissue expresses miRNA profiles that are distinct from normal tissue; therefore, the experimental design should ensure that the obtained data corresponds to each type of tissue.

### 4.4. Relevance of the Normal/Healthy Controls Used in These Studies

Only three studies that analyzed miRNAs in peripheral blood, serum or plasma of healthy subjects as controls provided details on how the “healthy” status of those subjects was verified [73,79,85]. This relevant information should have been included in the other studies. On the other hand, a few studies used subjects who suffered from disease as “normal controls”. For example, patients with pleural effusion were used as controls, which implies that these subjects suffered from a disease that was not disclosed in the study. Another example was the use of patients with coronary artery disease as normal controls. This scenario may be considered an important flaw in the design if the study aim was to compare MPM patients to “healthy” subjects. Evidence indicates that miRNAs are altered in several diseases; therefore, there is bias in analysis when the presence of another disease is not acknowledged.

### 4.5. Relevance of Proper Normalizers for Quantitative RT-qPCR Analysis (Validation Phase)

Currently, the relevance of proper and rigorous normalization of quantitative RT-qPCR data is well known. The accuracy of expression measurements requires the identification and validation of appropriate reference miRNA for each type of biological sample used [92,93,94]. It is notable that only a few studies identified and experimentally validated the most stable miRNAs to normalize qPCR expression data (Table 1). Inappropriate normalization can result in statistical confidence in the wrong conclusion [92] and can lead to false discovery.

### 4.6. Analysis of Different Histological Subtypes of MPM

In addition to different histological characteristics, there are differences in the clinical behavior, malignity and outcome between the three main histological subtypes of MPM. Consequently, analysis of miRNA expression may be separately performed for each histological subtype of MPM in order to maximize discoveries with clinical usefulness. Unfortunately, most studies did not report such analysis despite using different histological subtypes of MPM, or they omitted this analysis from the report it if it was actually performed. Moreover, some studies did not report the histological subtype of the samples used.

### 4.7. More Than One Biomarker Used for Diagnosis

Cancer, including MPM, is a multifactorial disease that involves multiple genetic/epigenetic alterations and environmental risk factors. Thus, it is unlikely that a single biomarker will provide a method of detection with the sensitivity and specificity required to reach an accurate diagnosis. Accordingly, evidence showed that the diagnostic value of serum levels of miR-126 increased when it was measured together with SMRPs and Met-TM [73,85], and the diagnostic value of levels of miR-103a-3p in the cellular fraction of peripheral blood increased when it was measured together with mesothelin in plasma [71,72]. In addition, signatures of two or more miRNAs may increase diagnostic value in future clinical use [87].

### 4.8. Complete and Accurate Reporting

Complete and accurate reporting allows readers to critically identify the strengths and weaknesses of the research study and therefore evaluate the validity and potential applicability of the reported data. However, critical information is often missing or unclear in most of the reviewed publications. This scenario is not unique for studies regarding MPM; lack of relevant information in design, conduct and analysis of diagnostic studies has been detected previously [95,96]. Because of this lack of information, the Standards for Reporting of Diagnostic Accuracy (STARD) initiative emerged to improve the quality of reporting of studies of diagnostic accuracy, among other guidelines. Recommendations of guidelines, such as the Standards for Reporting of Diagnostic Accuracy (STARD) for diagnostic studies [97], the Reporting recommendations for tumour Marker prognostic Studies (REMARK) for prognostic studies [98], or the Strengthening the Reporting of Observational Studies in Epidemiology (STROBE) for observational studies [99], are useful to determine the reliability and quality of biomarkers in the initial discovery phase. These guidelines have been available since 2003, 2007 and 2012, respectively. Following the recommendations of these guidelines may be helpful to standardize which vital information is published.

## 5. miRNAs Associated with Neoplastic Mechanisms of MPM and Their Potential Diagnostic Value

In addition, increasing evidence suggests that aberrant levels of miRNAs contribute to oncogenesis, progression and metastasis of several cancers, such as tumor suppressor or oncomiR [100]. As previously mentioned, the potential association of altered miRNAs with MPM carcinogenic mechanisms increases the likelihood of having a diagnostic value for this disease. This feature, together with their tumor tissue-specific expression, may facilitate the identification of diagnostic and prognostic miRNA biomarkers that can be applied for clinical use. Table 3 summarizes deregulated miRNAs associated with carcinogenesis mechanisms in MPM.

For example, miR-145 was reported as downregulated in MPM tissue by three independent studies; importantly, its over-expression in MPM cell lines induces a reduction of proliferation and migration in two out of three transfected MPM cell lines (Table 2 and Table 3).

Perhaps, greater effort should be devoted to elucidate which miRNAs are associated with neoplastic mechanisms along with the searching for candidate biomarkers.

## 6. Conclusions and Future Perspectives

Accurate diagnosis of MPM is often difficult and complex. Difficulty in diagnosis has led to the search for new diagnostic tools that can be added to the resources currently used in the clinic. In this regard, accumulating evidence indicates that miRNAs are potential diagnostic biomarkers for several tumors, which prompted the study of microRNA expression levels as an important diagnostic and prognostic tool for MPM.

However, for this disease, limited availability of patient cohorts seemed to be an initial problem that had to be solved in order to perform research. Possibly, in order to advance in the field, it would be preferable to identify the most promising candidate miRNAs reported in the peer-reviewed literature and validate them in a multi-institutional/international coordinated effort using well-characterized biological samples from multiple research institutions in statistically well-powered prospective studies.

However, analysis of the published literature showed the heterogeneity of the data, samples, controls, and methods and the critical limitations and potential bias of several of the reviewed studies. Moreover, important information is often missing or unclear in various revised papers. Nevertheless, despite these limitations, several common candidate biomarker miRNAs were confirmed by various studies. Moreover, some of these miRNAs were associated with cellular mechanisms that are potentially involved in carcinogenesis in in vitro experiments. This result is telling of the true potential of miRNAs as diagnostic biomarkers for MPM.

This analysis allows some essential conclusions. (A) Larger and prospective studies are needed to confirm the true diagnostic value of all candidate miRNAs reported in the reviewed literature. (B) It is fundamental that research is reported clearly and transparently regarding study design, performance, and analysis. (C) It is necessary to critically evaluate the published data in order to identify deficiencies or bias and to overcome these issues in future or subjacent studies. (D) To date, none of the studies have successfully reached the final objective, which is the use of miRNAs as diagnostic biomarkers in the clinic.

Finally, miRNA-based biomarker tests could add relevant adjunct information to increase the probability of reaching the right diagnosis. Therefore, they may be used as complementary tests to gold standard immunohistochemical diagnostic tests, X-rays and clinical data.

## Figures and Tables

**Table 1:** Studies regarding tissue and secreted miRNAs differentially expressed in MPM with potential diagnosis biomarker value.

Studies in	Sample Source	Study Design and Sample Size	Assay (and Number of miRNAs Analyzed)	miRNAs Differentially Expressed in MPM	ROC Analysis	Study Aim	Selection of Endogenous Stable Normalizer (Validation Phase)	Reference
	Pleural tissue (frozen)	Discovery: 17 MPM pleural tissue vs. 1 total RNA from normal human pericardium	Microarray (723)	let-7b*↑, MiR-1228*↑, miR-195*↑, miR-30b*↑, miR-32*↑, miR-345↑, miR-483-3p↑, miR-584↑, miR-595↑, miR-615-3p↑, and miR-885-3p↑. let-7e*↓, miR-144*↓, miR-203↓, miR-340*↓, miR-34a*↓, miR-423↓, miR-582↓, miR-7-1*↓ and miR-9↓	Not performed (NP)	Oncogenic mechanisms	NA	[58]
Tissue	Pleural and lung tissue	Discovery: 15 MPM pleural tissues vs. 10 AD pleural tissues. Validation: 100 MPM pleural tissues vs. 32 AD lung tissues	Microarray (2564), RT-qPCR (7)	RT-qPCR: miR-200c↓, miR-200b↓, miR-203↓, miR-141↓, miR-429↓ and miR-205↓	Specificity and sensitivity >80%	Diagnosis	NP. Use of RNU44 and RNU48	[59]
	Pleural and several tumor tissue (FFPE)	Discovery: 7 MPM pleural tissues vs. 97 epithelial carcinomas. Validation: (1) 32 pleura tissues MPM vs. 113 epithelial carcinomas. (2) 16 MPM pleural tissues vs. 23 epithelial carcinomas. (3) 14 pleural tissues MPM vs. 46 epithelial carcinomas	Microarray (747), RT-qPCR (3)	RT-qPCR: miR-193a-3p↑, miR-200c↓ and miR-192↓	Specificity 94%, sensitivity 100%	Diagnosis	NP. Use of U6 snoRNA	[60]
	Pleural tissue (frozen), pleural tissue (FFPE).	Discovery: 10 MPM vs. 5 Healthy controls (frozen). Validation: 27 MPM vs. 27 adjacent normal pleural tissues (FFPE)	PCRArray (88). RT-qPCR (3)	miR-126↓	NP	Diagnosis	NP. Use of U6 small nuclear RNA	[29]
	Pleural tissue (frozen)	Discovery: 25 MPM vs. 6 normal parietal pleura (patients without cancer). Validation: Same cohort? Not specified	Microarray (1145), RT-qPCR (4)	RT-qPCR: miR-206↓, miR-1↓, miR-483-5p↓ and miR-155*↑	NP	Oncogenic mechanisms	NP. Use of RNU44	[62]
	Pleural tissue (FFPE). Also MPM cell lines.	Discovery: [74]. Validation: 60 MPM vs. 23 normal pleural tissues	RT-qPCR (4)	miR-16↓, miR15a↓, miR-15b↓, and miR-195↓	NP	New therapy targets	NP. Use of RNU6B	[63]
	Pleural tissue (fresh, frozen & FFPE). Peritoneal tissue (frozen and FFPE).	Discovery: 29 MPM pleural tissues vs. 12 peritoneal mesothelial cysts (FFPE). Validation: (1) 6 MPM pleural tissues vs. 14 benign pleural tissues (Fresh). (2) 36 pleural tissues MPM vs. 36 peritoneal mesothelium (frozen)	Microarray (887), RT-qPCR (1)	RT-qPCR: miR-145↓	NP	Oncogenic mechanisms	NP. Use of RNU6B and RNU49	[64]
	Pleural tissue (FFPE)	Discovery: 5 MPM pleural tissues vs. 5 non-cancerous/non-inflammatory pleural tissues vs. 4 pleural chronic inflammation tissues vs. 5 mesothelial hyperplasia tissue	PCRArray (667)	miR-517b-3p↓, miR-627↓, miR-766-3p↓, miR-101-3p↓, miR-501-3p↓, miR-212-3p↓, miR-596↓, miR-145-5p↓, miR-671-3p↓, miR-181a-5p↓, miR-18a-3p↓, miR-30e-3p↑, miR-34a-3p↑, miR-622↑, let-7-g-5p↑, miR-196b-5p↑, miR-135b-5p↑, miR-18a-5p↑, miR-302b-3p↑	NP	Oncogenic mechanisms	Normalization factor: the global mean expression value	[65]
	Pleural tissue (FFPE)	Discovery: 5 preoperative pleural tissues with MPM (before Cth = DB) and 5 pleural tissues MPM (after Cth = MPMc) vs. 5 non-neoplastic pleura tissues (after Cth = NNP). Validation: 40 MPMc vs. 12 DB vs. 14 NNP	PCRArray (742), RT-qPCR (4)	RT-qPCR: miR-126↓*, miR-143↓, miR-145↓, miR-652↓	Specificity and sensitivity close to or >80%	Diagnosis	NP. Use of snord49A	[66]
	Pleural tissue (frozen)	Discovery: 18 MPM pleural tissues vs. 6 pleural tissues from benign asbestos-related pleural effusion patients (BAPE) (tissue with unspecific pleuritis/fibrosis)	PCRArray (384)	miR-484↑, miR-320↑, let-7a↑, miR-125a-5p↑	Specificity and sensitivity close to or >80%	Diagnosis	NP. Use of U6 snoRNA	[67]
	Pleural tissue (FFPE) and cells from pleural effusion.	Discovery and Validation: 17 MPM pleural tissues vs. 6 normal pleural tissues patients without cancer undergoing cardiac or aortic surgery. Cells from pleural effusion of 26 MPM patients vs. 10 benign pleural diseases	RT-qPCR (1)	miR-223↓	NP	Oncogenic mechanisms	NP. Use of RNU6B for tumor and RNU48, RNU44, or SNOR202 for cells	[68]
	Cell lines, pleural tissue (FFPE), pleural citology	Discovery: 2 MPM cell lines vs. 1 mesothelium cell line. Validation: (1) 51 MPM pleural tissues vs. 40 benign/reactive pleurae. (2) 29 MPM cytologic specimens vs. 24 reactive mesothelial cells	RT-qPCR (15)	miR-19a↑, miR-19b↑, miR-25↑, miR-21↑, miR-126↓	Specificity and sensitivity >80%	Diagnosis	NP. Use of RNA U6B	[69]
	Pleural tissue (FFPE), pleural citology	Discovery: Bionformatic analysis 3 database. Validation: 41 epithelioid MPM vs. 40 AD and 26 cytologic specimen epitheloid MPM vs. 26 AD	RT-qPCR (6)	miR-130a↑(histological and cytological specimens)	Specificity 67% and sensitivity 77%	Differential diagnosis MPM vs. AD	NP. Use of RNU6B	[70]
Peripheral blood	Cellular fraction of peripheral blood	Discovery: 23 MPM vs. 17 asbestos-exposed controls (AE). Validation: 23 MPM vs. 17 AE vs. 25 healthy controls	Microarray (328), RT-qPCR (2)	RT-qPCR: miR-103↓	Specificity and sensitivity >80%	Diagnosis	Yes: miR-125a	[71]
	Cellular fraction of peripheral blood	Discovery: [71].Validation: 43 MPM vs. 52 asbestos-exposed controls	RT-qPCR (1)	miR-103a-3p↓ (plus mesothelin↑ in plasma)	Specificity and sensitivity >80%	Diagnosis	Yes: miR-125a	[72]
Serum and plasma	Serum	Discovery: In pleural tissue (miR-126↓ same paper). Validation: 44 MPM vs. 196 asbestos-exposed controls vs. 50 Healthy controls	RT-qPCR (1)	miR-126↓	Specificity 74% and sensitivity 73%.	Diagnosis	NP. Use of U6 snoRNA	[29]
	Serum	Pre-Validation: [29]. Validation: 45 MPM vs. 20 NSCLC vs. 56 healthy controls	RT-qPCR (1)	miR-126↓	Specificity 60% and sensitivity 80%	Diagnosis	Yes: U6 snoRNA and use of exogenous control cel-miR-39	[73]
	Plasma, serum. Also pleural tissue (FFPE)	Discovery: 5 MPM (plasma) vs. 3 healthy controls (HC). Validation: 15 MPM (plasma) vs. 14 HC. Validation serum: 30 MPM (serum) *vs*. 10 asbestosis (serum). Validation tissue: 18 MPM pleural tissues vs. 7 pericardial tissues	Microarray (854), RT-qPCR	RT-qPCR: miR-625-3p↑ (plasma & serum). miR-625-3p↑, miR-29c*↓, miR-16↓, miR-196b↓, miR-26a-2-3p↓ and miR-1914-3p↓ (tissue)	Specificity & sensitivity close to or >80 % (plasma & serum miR-625-3p)	Diagnosis	Only SD of Cq range values without specified clearly which samples were used. Previous work (plasma): miR-16	[74]
	Serum	Discovery: 11 MPM (epithelial) vs. 45 healthy controls vs. 36 AD	Deep sequencing (Ilumina)	MPM vs. control: miR-4791↑, miR-185-5p↑, miR-96-5p↑, miR-1271-5p, miR-1292-5p↑, miR-409-5p↑ y miR-92b-5p↑	NP	Diagnosis	NA	[79]
	Serum	Discovery: 14 MPM vs. 10 non-cancer related effusions patients. Validation: Not specified	PCRArray (384), RT-qPCR (7)	RT-qPCR: miR-101↑, miR-25↑, miR-26b↑, miR-335↑, miR-29a↑, miR-516↑, miR-433↑, miR-191↓, miR-223↓	Not performed	Prognosis	NP. Use of miR-16	[80]
	Serum	Discovery: [29]. Validation 1: 45 MPM vs. 99 asbestos-exposed subjects (AE) vs. 44 healthy subjects. Validation 2:18 MPM vs. 50 (AE) vs. 20 healthy controls and 42 lung cancer (LC) patients	RT-qPCR (1)	Combination SMRPs↑, miR-126↓ and Met-TM↑	AUC of 0.857 (95% CI, 0.767–0.927)	Diagnosis	NP. Use of U6 snoRNA. Use of exogenous control cel-miR-39	[85]
	Serum	Discovery: 10 MPM vs. 10 asbestos-exposed subjects (AE) vs. 10 healthy controls (HC). Validation: 20 MPM vs. 15 AE vs. 14 HC	Microarray (1201), RT-qPCR (3)	RT-qPCR: miR-197-3p↑, miR-1281↑, miR-32-3p↑ (MPM vs. HS and MPM vs. AE)	AUC 95% CI, 0.5398-0.8959 (miR-197-3p)	Diagnosis	Yes: miR-3665	[86]
	Plasma (exosomal fraction)	Discovery: 23 MPM vs. 19 past asbestos-exposed subjects. Validation: Same samples minus 4	OpenArray (754). RT-qPCR (20)	2-miRNAs signatures: miR-103a-3p↓, miR-30e-3p↓	Specificity 80% and sensitivity 95.5%	Diagnosis	RNU48. It is not clear	[87]
	Plasma	Discovery: 21 MPM vs. 21 asbestos-exposed controls. Validation: 22 MPM vs. 44 asbestos-exposed controls	PCRArray (377), RT-qPCR (2)	RT-qPCR: miR-132-3p↓	Specificity 61% and sensitivity 86%	Diagnosis	Yes. Use of miR-146b-5p. Another untested normalizer was used too	[88]

AD = Lung Adenocarcinoma; Cth = chemotherapy; Met-TM = methylated thrombomodulin promoter; SMRPs = soluble mesothelin-related peptides. snoRNAs: small nuclear RNA; SD: standard desviation; NA = does not apply; NP = not performed. ROC = Receiver operating characteristic. ↑ = upregulated expression; ↓ = downregulated expression.

**Table 2:** miRNAs with potential diagnosis value for MPM reported by at least 2 independent studies.

miRNA	Number of Studies	Sample Source	Comparative Analysis Design	References
miR-200c↓	2	Pleural tissue	(1) MPM vs. AD. (2) MPM vs. epithelial carcinoma	[59,60]
miR-126↓	3	Pleural tissue	(1) MPM vs. normal pleura. (2) MPM vs. normal pleura (with Cth). (3) MPM vs. benign/reactive pleurae	[29,66,69]
miR-145↓	3	Pleural tissue	(1) MPM vs. benign pleural tissue. (2) MPM vs. normal pleura. (3) MPM vs. normal pleura (with Cth)	[64,65,66]
miR-16↓	2	Pleural tissue	(1) MPM vs. pericardial tissues. (2) MPM vs. normal pleural tissue	[63,74]
miR-103↓	2	Cellular fraction of peripheral blood	Two subsequent studies: MPM vs. asbestos-exposed controls	[71,72]
miR-126↓	3	Serum	Three subsequent studies: (a) MPM vs. asbestos-exposed controls vs. healthy controls. (b) MPM vs. Healthy controls. (c) MPM vs. asbestos-exposed controls vs. healthy controls	[29,73,85]

Only validated miRNAs (RT-qPCR) are included in this list. ↑ = upregulated expression; ↓ = downregulated expression.

**Table 3:** Deregulated miRNAs associated with carcinogenesis mechanisms in MPM.

miRNA with Deregulated Expression in MPM	Potential Function	Biological Effect of Experimental Manipulation of miRNA Expression	Other Effects	Reference
miR-16↓ (tissue)	Tumor suppressor	Restoring miR-16: growth inhibition, cell cycle arrest in G0/G1, increased apoptosis and reduced colony formation in MPM cell lines	Correlation with downregulation of Bcl2, CCND1. Administration of miR-16-containing minicells to xenografted mice inhibited tumor growth	[63]
miR-1↓ (tissue)	Tumor suppressor	Restoring miR-1: cell cycle arrest, increased apoptosis.	Correlation with upregulation of p53, BAX, p16/21; and downregulation of Bcl2 and survivin	[62]
miR-145↓ (tissue)	Tumor suppressor	Restoring miR-145: reduction of proliferation and migration of two out of three transfected MPM cell lines	Xenotransplant (transfected MPM cell line): inhibition of tumor growth in 6 of 8 treated mice compared to controls	[64]
miR-223↓ (tissue)	Tumor suppressor	Over-expression of miR-223: reduction of two MPM cell lines motility.	STMN1 levels were reduced and tubulin acetylation was induced	[68]

↑ = upregulated expression; ↓ = downregulated expression.
